# Effects of frequent, short-duration web-based light-intensity aerobic dance exercise on body composition, physical function, and physical activity in older adults: a randomized controlled trial

**DOI:** 10.1186/s12877-025-06495-3

**Published:** 2025-11-03

**Authors:** Yuya Watanabe, Kazuki Hyodo, Daisuke Yamaguchi, Takayuki Noda, Sumiyo Nishida, Aiko Ueno, Yuko Kai, Takashi Arao

**Affiliations:** 1 Physical Fitness Research Institute, Meiji Yasuda Life Foundation of Health and Welfare, Tokyo Hachioji, Japan; 2 Biwako Seikei Sport College, Faculty of Sport Study, Shiga Otsu, Japan

**Keywords:** Low-intensity exercise, Light physical activity, Web-based exercise, Online intervention, Randomized controlled trial, Gait speed, COVID-19

## Abstract

**Background:**

Physical inactivity remains a critical issue among older adults worldwide, contributing to functional decline and increased health risks. Traditional exercise programs face barriers such as accessibility and motivation. Web-based home exercise programs offer a scalable and accessible solution. This study examined the effects of a 12-week web-based, short-duration, and high-frequency light-intensity aerobic dance exercise program on body composition, physical function, and physical activity levels in older adults.

**Methods:**

Eighty-one older adults were randomly assigned to an exercise group (*n* = 41) or control group (*n* = 40). The exercise group participated in a 12-week home-based online program consisting of 20-minute light-intensity aerobic dance sessions held on weekday mornings. The control group maintained their usual lifestyle. Body composition, physical function, and physical activity levels were assessed before and after the intervention.

**Results:**

Seventy-one participants (36 in the exercise group and 35 in the control group) completed the study. The exercise group significantly improved the maximal walking speed by 0.10 m/s compared with the control group, with no significant between-group differences in body composition, physical activity levels, and other physical function outcomes. The adherence rate was 94.5%, indicating high feasibility of the intervention.

**Conclusions:**

The 12-week web-based aerobic dance exercise program selectively improved walking speed, suggesting its potential to enhance a specific aspect of locomotor function in older adults. Given their accessibility and scalability, web-based interventions may help in promoting functional independence and healthy aging. Future research should explore the long-term effects and optimize the program to maximize its impact.

**Trial registration:**

This study was registered in the UMIN Clinical Trials Registry (UMIN000044758, registered on 5 July 2021).

**Supplementary Information:**

The online version contains supplementary material available at 10.1186/s12877-025-06495-3.

## Background

 Physical activity is known to have a positive impact on various health outcomes in older adults, including physical, mental, and social functions [1, 2]. Conversely, sedentary behavior is associated with adverse effects on these outcomes [3]. Based on these findings, the World Health Organization (WHO) recommends regular moderate-to-vigorous intensity aerobic and muscle-strengthening physical activity along with reduction in sedentary behavior [4]. However, many older adults struggle to meet these guidelines due to physical limitations, pain, low motivation, or environmental barriers [5, 6]. Light-intensity physical activity (LPA) is a feasible and safer alternative for many older adults because it can be performed with lower physical or psychological stress, carries a reduced risk of injury, and can be easily integrated into daily routines [7]. Emerging evidence from intervention and observational studies suggests that LPA can improve physical function—such as mobility, balance, and strength—as well as cardiovascular and metabolic health [7–9].

Furthermore, internet-based interventions that deliver exercise programs to the home have gained attention as a promising way to overcome environmental barriers, such as transportation difficulties or lack of access to facilities [10]. The adoption of these online programs was significantly accelerated by the COVID-19 pandemic, which restricted in-person activities [10]. Recent studies have investigated the feasibility of various home-based online exercise programs such as resistance training, aerobic exercise, and yoga [11–14]. Granet et al. [14] conducted a 12-week intervention in which older adults were randomly assigned to either a live interactive exercise group or recorded exercise group. Both groups showed significant improvements in physical fitness measures such as walking speed and the five-repetition sit-to-stand test. Notably, the live interactive group exhibited greater improvements and a lower dropout rate (16%) compared to the recorded group (46%). These results suggest that real-time interactions with the instructors and other participants are crucial for maintaining motivation and adherence to exercise programs.

Therefore, to create an exercise program that is both feasible and accessible for older adults, we developed a web-based, short-duration (20-minute), light-intensity aerobic dance exercise program delivered via videoconferencing platforms, such as the Zoom application. Our previous pilot study confirmed the feasibility, safety, enjoyment, and usability of this program [15], suggesting that the program could contribute to health promotion and frailty prevention among older adults. However, its effects on body composition, physical function, and physical activity levels remain unclear. This study addresses gaps in the existing literature by evaluating, for the first time, a short-duration (20 min), high-frequency, light-intensity, interactive online exercise program for older adults in a randomized controlled trial, with multiple outcomes including body composition, physical function, and objectively measured physical activity. Evidence on the effectiveness of such light-intensity online programs for older adults who face various barriers to engaging in moderate-to-vigorous physical activity remains limited.

The present study, therefore, aimed to evaluate whether participation in a short-duration, high-frequency, 12-week online aerobic dance exercise intervention on weekday mornings could lead to improvements in body composition, physical function, and daily physical activity levels among community-dwelling older adults. Given that this trial primarily aimed to examine the efficacy of the program, all participants in the intervention group were provided with the necessary ICT support system, including devices and internet connectivity, to minimize technological barriers. We hypothesized that participation in the program would (1) increase daily physical activity due to the energizing effects of morning exercise, (2) positively influence body composition by increasing the lean muscle mass and reducing the fat mass, and (3) enhance physical function, such as balance and mobility, by promoting more functional body movements.

## Research design and methods

### Participants

The participants in this study were recruited from community-dwelling older adults aged 60 years or older living in Hachioji City, Tokyo, Japan using flyers. Cognitive function was screened using the Japanese version of the Mini-Mental State Examination (MMSE-J) [16], with a cut-off score of ≥ 24 points. The MMSE-J was administered with official forms obtained from an authorized distributor. Medical history and medication status, including any prior diagnosis of dementia or stroke, were investigated using a self-reported questionnaire. Exclusion criteria were as follows: (1) restriction of exercise by a home doctor; (2) inability to exercise in a standing position; (3) history of stroke, dementia, or serious illness; (4) MMSE-J score of 23 or lower; (5) use of any tranquilizer or hormonal medication; and (6) uncontrolled hypertension (systolic blood pressure ≥ 160 mmHg and/or diastolic blood pressure ≥ 95 mmHg). Participants were randomly assigned to groups using stratified randomization based on the sex and age category (under 75 years and 75 years or older) to ensure balance between groups.

All participants were fully informed of the purpose, procedures, and risks of this study, and provided written informed consent before participation. The Ethics Committee of the Physical Fitness Research Institute of the Meiji Yasuda Life Foundation of Health and Welfare approved the study protocol (approval number: 2022-0001). This study has been registered in the Clinical Trials Database (UMIN000044758).

### Study procedure

This randomized controlled trial of a 12-week web-based exercise intervention was conducted in two separate cohorts: from September to November 2021 and from January to April 2022. After the pre-intervention measurements, the participants were randomly allocated to the exercise and control groups. The exercise group participated in a web-based exercise session held on weekday mornings for 12 weeks. The control group was instructed to maintain a normal lifestyle for 12 weeks. Post-intervention measurements were conducted after the 12-week intervention period.

### Intervention program

The details of this intervention program have been previously reported [15]. Briefly, the online exercise program was delivered via Zoom and led by one of five professional instructors. Each session lasted 20 min and included a 5-minute warm-up, 10 min of light-intensity aerobic dance (“Slow Aerobic^®^”), and a 5-minute cool-down. The program was conducted five days per week (Monday to Friday) for 12 weeks. Participants could choose between two morning sessions (8:30 AM or 9:30 AM). One of the five professional aerobic dance exercise instructors (one man and four women) led the exercise program using Zoom.

The Slow Aerobic program is a light-intensity, rhythmical aerobic dance exercise designed specifically for older adults. It incorporates three key movement patterns: (1) lateral trunk extensions, (2) chest-opening arm movements, and (3) trunk rotations. Detailed movement descriptions are provided in [17] for detailed movement descriptions. These movements are performed to music at a tempo of 90–120 beats per minute, selected to ensure safe and comfortable participation for older adults [17, 18]. The program emphasizes dynamic trunk and thoracic mobility to promote flexibility, postural control, and overall physical activity. Exercises are performed while standing, without the need for specialized equipment or large space, making the program accessible and easy to implement at home. To maintain engagement, the routine was slightly modified every two weeks.

### Online exercise delivery and safe management system

To facilitate participation, each participant received the necessary devices, including a tablet pre-installed with the required applications (Zoom and LINE WORKS), a heart rate monitor, a smartphone for heart rate tracking, and a Wi-Fi router to ensure stable internet connectivity [15]. All devices were loaned free of charge, and the participants were provided with step-by-step guidance on using the equipment. Daily reminders with the session link were sent through LINE WORKS each morning, simplifying the process of joining the exercise sessions.

For safety management, participants were required to complete a brief health status checklist via LINE WORKS before each session. This included self-reported blood pressure and overall health condition. Participants with a systolic blood pressure of 180 mmHg or higher or diastolic blood pressure of 110 mmHg or higher were advised to refrain from participating that day for safety reasons. During the sessions, the participants’ heart rates were monitored in real time using a heart rate monitor (OH1, Polar, Finland), with data transmitted to the research team through a web-based system. The research staff continuously observed the participants’ movements via Zoom to ensure proper exercise techniques and promptly addressed any concerns.

### Measurements

Anthropometric indices, body composition, physical function, and physical activity were measured before and after the 12-week intervention at a community center or our research institute. Six trained members of our laboratory performed the measurements. Height was measured as the length from the bottom of the foot to the top of the head to the nearest 0.1 cm using an analog stadiometer. Weight, fat-free mass, and fat percentage were measured using a bioelectrical impedance device (InBody 430; Biospace, Korea) while participants were barefoot. Body mass index (BMI) was calculated by dividing weight by the square of height.

We conducted a battery of physical function tests including grip strength, chair standing, one-leg standing with eyes open, and normal and maximal walking speeds. Grip strength was measured using a Smedley hand dynamometer (TKK5401, Takei Scientific Instruments, Japan) [19, 20]. Two trials with maximum effort, separated by a brief rest period, were performed separately for each hand. The maximum value of the four trials (two trials for each hand) was used for the analysis. The five-time chair standing and 30-second chair standing tests were conducted [19, 20]. The participants were instructed to stand up and sit down as quickly as possible on a firm, padded, and armless chair. The participants’ performance in the five-time sit-to-stand time was measured using a stopwatch, and the number of repetitions within 30 s was counted. Each test was performed once. The standing time on one foot with the eyes open was measured using a stopwatch [19]. Participants were instructed to stand on any one foot with hands on the hips. The examiner explained to the participants that the measurement would be stopped if the standing foot moved from the starting position, if the raised leg touched the floor or the standing foot, or if the hands on the hip became detached. The maximum measurement time was 180 s. Two trials were performed separated by a brief rest period. The higher value was used for the analysis. The 10-m walking test was conducted at normal and maximal walking speeds, and the 6-m walking time, after excluding the first and last 2 m, was measured using a phototube system (Witty, Microgate, Italy). In this study, four pairs of photoelectric cells were placed at the starting line and at 2 m, 8 m, and 10 m from it. The participants were instructed to walk at their usual pace to assess habitual walking in the normal walking test, and to walk as fast as possible in the maximal walking test. In both cases, two trials were performed with a brief rest period in between, and the shorter walking time was used for the analysis. The walking speed (m/s) was calculated as 6 m divided by the shorter walking time from the two trials [19, 20].

A validated triaxial accelerometer (Active Style Pro HJA750-C, Omron Healthcare, Japan) was used to assess physical activity (PA) and sedentary behavior (SB) [21, 22]. Participants were instructed to wear the device on their hips during waking hours for at least 10 days. The epoch length was set to 60 s, and estimated metabolic equivalents (METs) were calculated using manufacturer-provided software. Non-wear time was defined as any continuous 60-minute period with activity counts below the detection limit [23]. A day was considered valid if the participant wore the accelerometer for at least 10 h [24], and only participants with at least four valid days were included in the analysis. Each 60-second epoch was categorized as SB (≤ 1.5 METs), light intensity physical activity (LPA, 1.6–2.9 METs), or moderate-to-vigorous intensity physical activity (MVPA, ≥ 3.0 METs) [25, 26]. The time spent on each activity was aggregated per day and averaged across all valid days.

### Statistical analysis

Statistical analyses were performed using R 4.4.1 (R Foundation for Statistical Computing, Vienna, Austria). To examine the effects of the intervention, we performed an analysis of covariance (ANCOVA) for all variables. The dependent variable was the post-test score, whereas the independent variable was the group (exercise vs. control). The baseline (pre-test) score was included as a covariate to control for baseline differences. For the main analysis, we conducted ANCOVA using the modified intention-to-treat (mITT) approach based on complete cases. For the sensitivity analyses, we conducted three additional analyses: (1) an ITT analysis using multiple imputations for missing data to assess the impact of selection bias (see Supplementary Table 1 for details); (2) mITT analysis that additionally adjusted for step count, beyond the baseline covariates used in the main analysis, to address potential confounding due to between-group differences; and (3) a per-protocol analysis.Statistical significance was set at p < 0.05.

## Results

**Fig. 1:**
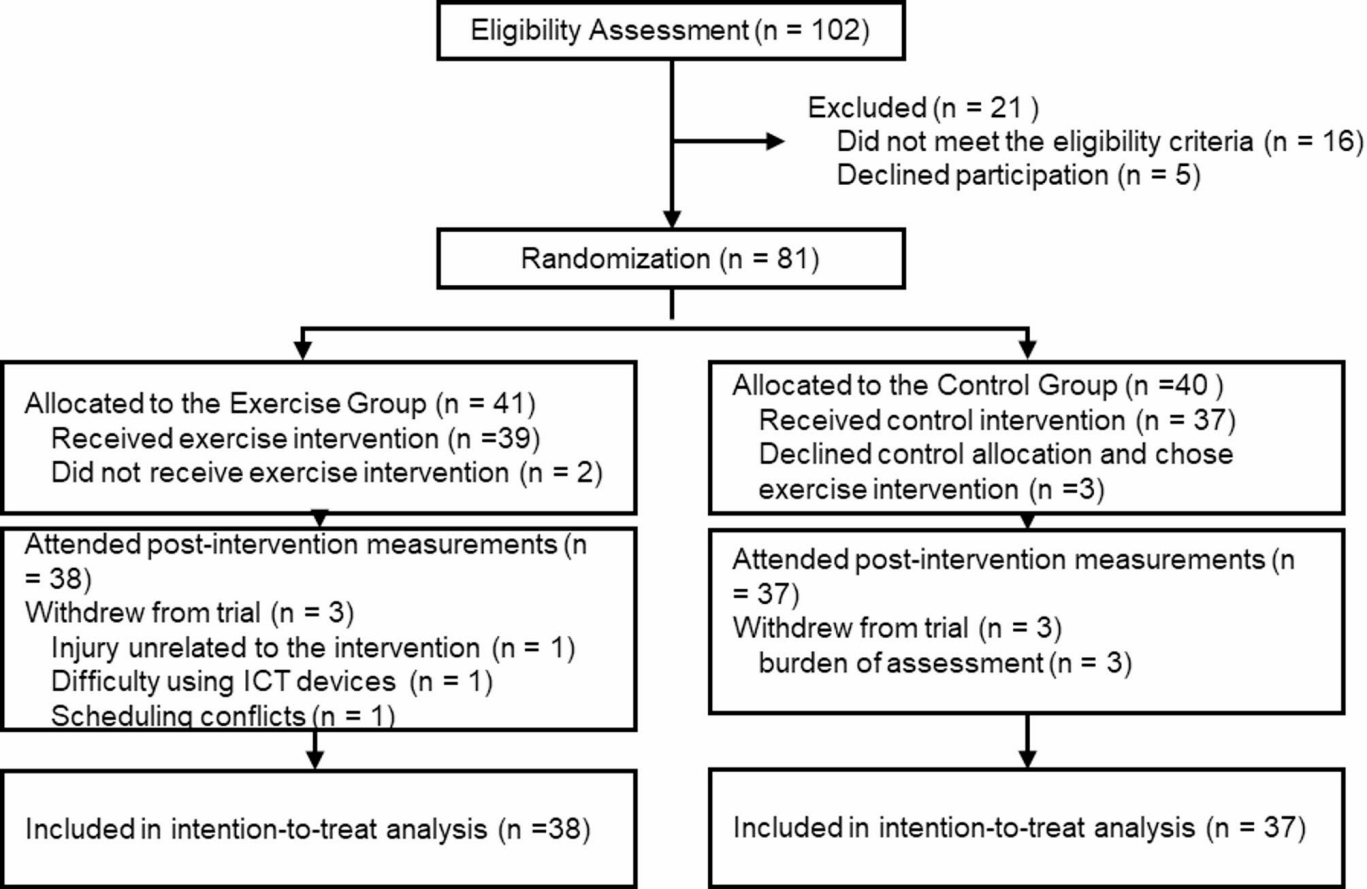
CONSORT flow diagram

The CONSORT diagram for this study is shown in Fig. 1. A total of 102 older adults participated in the baseline assessment (pre-measurement) for the 12-week web-based exercise intervention. Of these, 21 were excluded because they either did not meet the inclusion criteria (*n* = 16) or declined to participate (*n* = 5). Consequently, 81 participants were randomly assigned to either the exercise group (*n* = 41) or control group (*n* = 40). At the time of allocation, five participants (two in the exercise group and three in the control group) declined their assigned intervention, but participated in the post-measurement assessment. Additionally, six participants (three in the exercise group and three in the control group) withdrew from the study during the intervention period and did not participate in the post-measurement assessment. Reasons for withdrawal were intervention-unrelated injury (*n* = 1), difficulty in using ICT (*n* = 1), and schedule conflicts (*n* = 1) in the exercise group, and burden of assessment (*n* = 3) in the control group. No withdrawals were attributed to the intervention. Although not indicated in the CONSORT diagram, one participant in the exercise group experienced intervention-unrelated knee injury midway through the intervention, resulting in a significant decline in their participation rate; therefore, this participant was excluded from the per-protocol analysis. Thus, the main ITT analysis included data from 38 participants in the exercise group and 37 participants in the control group who participated in the post-intervention assessment. For the per-protocol analysis, 35 participants in the exercise group and 34 in the control group were included. Furthermore, for the multiple imputation analysis, missing values were imputed for all participants, allowing the analysis to include data from 41 to 40 participants in the exercise and control groups, respectively. Aside from the imputation analysis, the number of participants included in each analysis varied slightly by measure due to missing data on specific items. The demographic characteristics of both groups before the intervention are summarized in Table 1.

**Table 1:** Baseline characteristics of the participants

**Characteristic**	All (*n* = 81)	Exercise(*n* = 41)	Control(*n* = 40)
Age	75.9 (5.2)	76.0 (5.2)	75.8 (5.4)
Sex, male	13 (16.0%)	6 (14.6%)	7 (17.5%)
Education (years)	13.0 (2.1)	12.9 (2.0)	13.2 (2.2)
MMSE (score)	28.4 (1.7)	28.5 (1.5)	28.4 (1.9)
Self-rated health, ≥ Good	65 (80.2%)	36 (87.8%)	29 (72.5%)
Self-rated economic status, ≥ Good	69 (86.3%)	38 (92.7%)	31 (79.5%)
(Missing)		0	1
Living alone	18 (22.2%)	9 (22.0%)	9 (22.5%)
Internet use, Yes	63 (77.8%)	33 (80.5%)	30 (75.0%)
Exercise stage			
Precontemplation	1 (1.2%)	1 (2.4%)	0 (0.0%)
Contemplation	19 (23.5%)	6 (14.6%)	13 (32.5%)
Preparation	14 (17.3%)	6 (14.6%)	8 (20.0%)
Action	6 (7.4%)	3 (7.3%)	3 (7.5%)
Maintenance	41 (50.6%)	25 (61.0%)	16 (40.0%)
Clinical medication, Yes			
Hypertension	28 (34.6%)	12 (29.3%)	16 (40.0%)
Diabetes	5 (6.2%)	2 (4.9%)	3 (7.5%)
Alcohol intake			
Never	52 (64.2%)	25 (61.0%)	27 (67.5%)
1–3 day/month	6 (7.4%)	3 (7.3%)	3 (7.5%)
1–2 day/wk	11 (13.6%)	9 (22.0%)	2 (5.0%)
3–4 day/wk	4 (4.9%)	1 (2.4%)	3 (7.5%)
5-day/wk	8 (9.9%)	3 (7.3%)	5 (12.5%)
Smoking habit, current	3 (3.7%)	2 (4.9%)	1 (2.5%)
Volunteer groups			
Never	46 (56.8%)	26 (63.4%)	20 (50.0%)
1–3×/month	20 (24.7%)	10 (24.4%)	10 (25.0%)
1×/week	7 (8.6%)	3 (7.3%)	4 (10.0%)
2–3×/week	5 (6.2%)	1 (2.4%)	4 (10.0%)
≥4×/week	3 (3.7%)	1 (2.4%)	2 (5.0%)

**Characteristic**	All (*n* = 81)	Exercise(*n* = 41)	Control(*n* = 40)
Hobby groups			
Never	24 (29.6%)	10 (24.4%)	14 (35.0%)
1–3×/month	32 (39.5%)	17 (41.5%)	15 (37.5%)
1×/week	19 (23.5%)	10 (24.4%)	9 (22.5%)
2–3×/week	5 (6.2%)	3 (7.3%)	2 (5.0%)
≥4×/week	1 (1.2%)	1 (2.4%)	0 (0.0%)
Senior citizen clubs			
Never	56 (69.1%)	27 (65.9%)	29 (72.5%)
1–3×/month	18 (22.2%)	9 (22.0%)	9 (22.5%)
1×/week	3 (3.7%)	2 (4.9%)	1 (2.5%)
2–3×/week	2 (2.5%)	2 (4.9%)	0 (0.0%)
≥ 4×/week	2 (2.5%)	1 (2.4%)	1 (2.5%)
Neighborhood associations			
Never	51 (63.0%)	26 (63.4%)	25 (62.5%)
1–3×/month	28 (34.6%)	14 (34.1%)	14 (35.0%)
2–3×/week	2 (2.5%)	1 (2.4%)	1 (2.5%)
Learning/education circles			
Never	52 (64.2%)	24 (58.5%)	28 (70.0%)
1–3×/month	19 (23.5%)	11 (26.8%)	8 (20.0%)
1×/week	8 (9.9%)	6 (14.6%)	2 (5.0%)
2–3×/week	1 (1.2%)	0 (0.0%)	1 (2.5%)
≥ 4×/week	1 (1.2%)	0 (0.0%)	1 (2.5%)
Paid work			
Never	69 (85.2%)	37 (90.2%)	32 (80.0%)
1–3×/month	4 (4.9%)	2 (4.9%)	2 (5.0%)
1×/week	2 (2.5%)	0 (0.0%)	2 (5.0%)
2–3×/week	3 (3.7%)	1 (2.4%)	2 (5.0%)
≥ 4×/week	3 (3.7%)	1 (2.4%)	2 (5.0%)
Physical Characteristic & Body composition			
Height (cm)	153.6 (7.9)	153.4 (7.3)	153.8 (8.6)
Weight (kg)	53.4 (9.1)	52.6 (8.2)	54.3 (10.0)
Body mass index (kg/m2)	22.6 (3.4)	22.3 (2.8)	23.0 (4.0)
Muscle mass(kg)	19.1 (3.4)	18.8 (3.0)	19.4 (3.9)

**Characteristic**	All (*n* = 81)	Exercise(*n* = 41)	Control(*n* = 40)
(Missing)	3	1	2
Fat mass (kg)	17.2 (6.5)	16.9 (5.4)	17.5 (7.6)
(Missing)	3	1	2
Fat percent (%)	31.6 (8.1)	31.7 (6.9)	31.5 (9.3)
(Missing)	3	1	2
Physical function			
Grip strength (kg)	23.3 (6.1)	22.7 (5.6)	24.0 (6.5)
Five-times chair standing (s)	7.7 (2.0)	7.5 (2.0)	7.9 (2.0)
(Missing)	1	0	1
Chair standing for 30 s (numbers)	21.6 (5.5)	21.9 (5.1)	21.3 (6.0)
(Missing)	2	0	2
One leg standing with eyes open (s)	52.3 (53.9)	51.7 (50.8)	52.9 (57.6)
Normal walking speed (m/s)	1.37 (0.23)	1.37 (0.20)	1.38 (0.25)
Maximal walking speed (m/s)	1.82 (0.32)	1.83 (0.30)	1.82 (0.34)
Physical activity			
Daily step count (steps/day)	4,543 (2,197)	5,203 (2,400)	3,847 (1,735)
(Missing)	3	1	2
SB (%)	59.2 (9.8)	57.5 (11.4)	61.0 (7.5)
(Missing)	3	1	2
LPA (%)	36.6 (8.6)	37.7 (10.1)	35.5 (6.7)
(Missing)	3	1	2
MVPA (%)	4.1 (2.7)	4.8 (3.1)	3.4 (2.1)
(Missing)	3	1	2

Continuous variables are presented as mean (standard deviation), and categorical variables are as *n* (%)

*MMSE* mini-mental state examination, *SB *sedentary behavior, *LPA *light physical activity, *MVPA *moderate-to-vigorous physical activity

The retention rate in the exercise group was 92.3%, with three dropouts out of the 39 participants who initially accepted the allocation. The mean attendance rate of the 39 participants in the exercise group was 94.5 ± 6.4%. In addition, the mean heart rate during the exercise program was 90.6 ± 10.5 bpm, and the heart rate reserve (HRR) was 22.2 ± 11.5%. Based on the HRR, we confirmed, in agreement with previous studies, that the Slow Aerobic class is an exercise in the light-intensity range as defined by the ACSM (HRR less than 40% during exercise) [15]. No intervention-related adverse events were observed in this study.

Supplementary Figs. 1–3 presents the unadjusted individual pre- and post-intervention values for each measurement item in the exercise and control groups. Table 2 presents the ITT-based ANCOVA results, including the adjusted post-intervention values and statistical outcomes. Supplementary Tables 1–3 show the sensitivity analyses, including multiple imputation ITT, per-protocol analysis, and models adjusted for the step count.

**Table 2:** Post-intervention outcomes: adjusted means in the exercise intervention and control groups, and results of ANCOVA with baseline values as a covariate based on an intention-to-treat analysis

	Exercise group		Control group		Between-group adjusted mean difference (95% CI)	*p* value	d
	*N*	Adjusted mean (SE)	*N*	Adjusted mean (SE)			
Physical charactaristics							
Height (cm)	37	153.5 (0.1)	34	153.5 (0.1)	0 (-0.2, 0.2)	0.855	0.04
Weight (kg)	37	52.6 (0.2)	34	53.1 (0.2)	-0.5 (-1.2, 0.1)	0.102	-0.4
Body mass index (kg/m2)	37	22.3 (0.1)	34	22.5 (0.1)	-0.2 (-0.5, 0.1)	0.129	-0.37
Body composition							
Muscle mass (kg)	36	19.4 (0.1)	34	19.4 (0.2)	-0.1 (-0.5, 0.3)	0.693	-0.1
Fat mass (kg)	36	16 (0.3)	34	16.5 (0.3)	-0.5 (-1.3, 0.3)	0.214	-0.3
Fat percent (%)	36	29.9 (0.5)	34	30.3 (0.5)	-0.3 (-1.6, 1)	0.618	-0.12
Physical function							
Grip strength (kg)	37	23.9 (0.5)	34	23.6 (0.5)	0.3 (-1.1, 1.7)	0.695	0.1
Five-times chair standing (s)	37	7.4 (0.3)	34	7.3 (0.3)	0 (-0.9, 1)	0.957	0.01
Chair standing for 30 s (numbers)	37	22.8 (0.6)	33	22.1 (0.6)	0.7 (-1.1, 2.4)	0.452	0.18
One leg standing with eyes open (s)	37	70.5 (7.4)	34	58.5 (7.7)	11.9 (-9.5, 33.3)	0.27	0.27
Normal walking speed (m/s)	37	1.32 (0.02)	34	1.27 (0.02)	0.05 (-0.01, 0.12)	0.115	0.38
Maximal walking speed (m/s)	37	1.93 (0.03)	34	1.83 (0.03)	0.1 (0.01, 0.2)	0.033*	0.52
Physical activity							
Daily step count (steps/day)	37	4906 (195)	37	4743 (195)	163.1 (-399, 725)	0.565	0.14
SB (%)	37	55.5 (0.9)	37	57.6 (0.9)	-2.1 (-4.7, 0.4)	0.104	-0.39
LPA (%)	37	40.5 (0.8)	37	38.3 (0.8)	2.2 (-0.2, 4.5)	0.067	0.44
MVPA (%)	37	3.9 (0.2)	37	4.2 (0.2)	-0.2 (-0.9, 0.4)	0.477	-0.17

*SB* sedentary behavior, *LPA *light physical activity, *MVPA *moderate-to-vigorous physical activity

*p < 0.05. d = Cohen's d

After the 3-month intervention, the exercise group had a significantly higher maximal walking speed than the control group (*β* = 0.10, 95% CI [0.01, 0.2], *p* = 0.033, *d* = 0.52). Although not statistically significant, point estimates of LPA favored the intervention group (*β* = 2.2, 95% CI [−0.2, 4.5], *p* = 0.067, *d* = 0.44). These trends remained consistent across all sensitivity analyses. No significant differences were detected between the groups for the other variables.

## Discussion

This study investigated the effects of a 12-week web-based, short-duration, light-intensity aerobic exercise program on the body composition, physical function, and physical activity in community-dwelling older adults. Despite the program being held five times a week on weekdays, adherence rates remained remarkably high, with an average attendance rate of 94.5%. Additionally, dropouts due to difficulties with ICT devices were minimal, suggesting that the provided support system effectively facilitated participation. Regarding the effects of the intervention on the measured outcomes, although no significant changes were observed in other physical function measures, physical activity levels, or body composition, maximal walking speed significantly improved by a clinically meaningful 0.10 m/s in the intervention group compared to the control group [27, 28]. These results suggest that such programs are both feasible and potentially effective in enhancing specific aspects of locomotor function among older adults.

In this study, the intervention did not significantly increase daily physical activity levels, as measured by an accelerometer. This result suggests that engaging in the short-duration (20-min) exercise on weekday mornings may not be sufficient to significantly increase the overall volume of physical activity in daily life. However, the adjusted mean difference between the groups showed that LPA was approximately 2.1% (95% CI [−0.2, 4.5]) higher and SB was 2.1% (95% CI [−4.7, 0.4]) lower in the exercise group, corresponding to an average increase of about 20 min in the LPA and decrease of approximately 20 min in SB. A similar pattern was observed in the sensitivity analyses, further supporting the robustness of our findings. Given that the intervention involved 20 min of LPA every morning, it is possible that this intervention directly contributed to the observed changes in physical activity. Notably, previous studies have raised concerns that structured exercise interventions may lead to compensatory reductions in spontaneous daily activities during the rest of the day [29], the absence of such compensation in this study is noteworthy. Even LPA has been linked to improvements in physical [7, 30] and cognitive function [31, 32], and to a reduced risk of metabolic or cardiovascular diseases [8, 9]. Accordingly, the observed increase in LPA and decrease in SB in the intervention group may have meaningful long-term health benefits.

With regard to physical characteristics and body composition measures, there were no significant differences between groups. Given the limited duration and light intensity of the intervention, the exercise program was probably insufficient to induce significant changes in the body composition. However, in the per-protocol analysis, the exercise group showed slightly lower body weight (−0.6 kg, 95% CI [−1.3, 0.1]) and fat mass (−0.6 kg, 95% CI [−1.4, 0.2]) without decrease in muscle mass (0 kg, 95% CI [−0.5, 0.4]) compared to the control group. Although small, these shifts may reflect early physiological adaptations among participants with high adherence. This is particularly important for older adults because maintaining muscle mass while reducing fat plays a crucial role in preventing sarcopenia and preserving functional independence in this subpopulation [33, 34]. Future studies should examine whether longer interventions can produce more substantial effects.

The exercise group demonstrated a significant increase in maximal walking speed compared to the control group, with an adjusted post-intervention group difference of 0.10 m/s (95% CI [0.01, 0.20]). Additionally, although the difference in usual walking speed did not reach statistical significance, a similar trend was observed, with an adjusted post-intervention group difference of 0.05 m/s (95% CI [−0.01, 0.12]). Sensitivity analyses revealed comparable estimates, further supporting the robustness of these findings. These results indicate that the online aerobic dance program (Slow Aerobic) can improve locomotor function among older adults. Given that walking speed is a well-established predictor of physical function and mortality in older adults, improvement in walking speed can be clinically meaningful [35, 36]. An increase of ≥ 0.10 m/s in gait speed is widely regarded as a clinically meaningful difference for older adults [27, 28]. Such an improvement has been linked to reductions in all-cause mortality, alongside fewer disabilities and lower healthcare utilization/hospitalization [37–39]. Therefore, this threshold-level improvement may translate into meaningful health benefits. The average maximal walking speed of Japanese older adults is 1.77 m/s for those in their 70 s and 1.63 m/s for those in their 80 s, indicating a decline of 0.14 m/s over a decade [40]. Accordingly, the 0.10 m/s improvement observed in this study can be interpreted as a recovery equivalent to approximately 7.1 years of age-related decline. Improvements in maximal walking speed observed in this study may be partly attributed to enhanced trunk flexibility and postural control. The Slow Aerobic program emphasized dynamic movements of the spine and thoracic cage. Thoracic mobility and functional capacity are important for maintaining efficient gait patterns, particularly stride length and walking speed, in older adults [41, 42]. Additionally, repeated practice of coordinated whole-body movements to music may have supported improvements in balance and motor control—both of which are key determinants of safe and effective gait performance [43, 44]. These adaptations may have contributed to the improvement in maximal walking speed, even in the absence of significant changes in muscle strength.

In contrast, no significant changes were observed in muscle strength or static balance. This suggests that a more targeted program incorporating resistance or higher-intensity training may be necessary to improve strength and power [45]. Indeed, recent findings by Li et al. [46] indicate that high-intensity interval walking can improve physical functions compared to moderate intensity exercise in older adults, highlighting the critical role of exercise intensity in achieving physiological adaptations. Future studies should consider longer-term or more intensive programs to achieve greater effects on muscle strength and body composition.

Similar to our pilot study [15], the feasibility and adherence rates in this study were high, with an average attendance rate of 94.5%, indicating high participant commitment to the program. Notably, only one participant withdrew from the study because of difficulties with ICT devices, suggesting that the provided technological support effectively minimized the barriers to participation. This finding highlights the feasibility of implementing web-based exercise programs, even among older adults, who are often considered to face challenges when using digital tools. Real-time interaction with instructors and peers has been shown to enhance motivation and adherence. In contrast, recorded or asynchronous programs often have high dropout rates [14]. The structured and interactive nature of this intervention may have contributed to participant retention. Given the accessibility and scalability of web-based exercise programs, these findings suggest that such interventions could be effective strategies for maintaining functional independence and overall wellbeing in older adults, ultimately contributing to the extension of healthy life expectancy.

This study has several limitations. First, the study participants were older adults who voluntarily expressed willingness to participate in an online exercise program. In the exercise group, 78% reported regular Internet use, which is slightly higher than the national average of 67% for Japanese adults aged 70–79 years in 2023 [47]. This indicates that our sample may have been more digitally literate, health-conscious, and motivated than the general older population. Such characteristics could lead to higher observed participation rates and benefits than might be expected in the general older population. Therefore, the generalizability of our findings to the broader older adult population, particularly those with low ICT literacy or motivation, should be interpreted with caution. Second, although high adherence was observed in this study, the ICT devices and Wi-Fi were provided by our research team. While this ensured minimal technological barriers and allowed us to assess the program’s effects under optimal conditions, real-world implementation without such support may pose challenges, including the cost and availability of suitable devices, stable internet connectivity, and the need for technical assistance [48]. To support broader adoption, potential strategies include utilizing existing community centers to provide shared devices and internet access, offering brief user training before program initiation, and simplifying user interfaces for individuals with limited ICT experience. To enhance real-world scalability, we have piloted and are now implementing a group-based approach in community settings, in which older adults gather at local facilities to participate in real-time online sessions led by instructors. This model eliminates the need for personal ICT devices or home internet access, making the program more accessible to those with limited digital resources or skills. It may also foster social interaction and motivation through group participation. Additionally, we have developed a prototype of a home-based delivery method using smart TVs with Chromecast, allowing participants to join sessions via their television and remote control [49]. This approach may reduce equipment costs and improve usability. Third, the 12-week duration and light intensity of the intervention may have been insufficient to induce measurable changes in muscle strength or body composition. Future trials should investigate whether combining this type of light-intensity aerobic dance program with more intensive components (e.g., resistance or interval training) can produce greater benefits while maintaining feasibility and adherence in older adults. Fourth, maintenance of the observed effects was not assessed beyond 12 weeks. Future studies should explore strategies to support long-term maintenance of these benefits. Possible approaches include periodic booster sessions, continued group-based programs in community settings, or self-directed home exercises supported by digital platforms. Finally, the mechanisms underlying the improvement in walking ability with Slow Aerobic remain unclear. One possible reason is that functional domains such as flexibility and dynamic balance were not directly assessed in this study. The inclusion of such assessments (e.g., Trunk Rotation Test [50], Functional Reach Test [51], Two-Step Test [52], and Timed Up and Go Test [53]) in future research would help elucidate the physiological and biomechanical factors contributing to the observed improvements and provide a more comprehensive understanding of the impact of web-based exercise programs on overall physical function.

## Conclusions

This study demonstrated that a 12-week web-based, short-duration, light-intensity aerobic dance program significantly improved the maximal walking speed of older adults, indicating enhanced locomotor function in older adults. Given its high feasibility, accessibility, and scalability, this program can be a practical tool for maintaining functional independence in older adults. Exploring long-term effects in the real world and integrating resistance exercise components should be next steps for maximizing physiological benefits and supporting functional independence in aging populations. In addition, developing scalable delivery strategies—such as streaming via home televisions or implementing group-based sessions in local community centers—may further improve accessibility and facilitate the broader adoption of online exercise programs for older adults.

## Supplementary Information

Supplementary Material 1.

## Data Availability

All data sharing and collaboration requests should be directed to the corresponding author (k-hyodo@my-zaidan.or.jp).

## Abbreviations

ACSM: American college of sports medicine
ANCOVA: Analysis of covariance
BMI: Body mass index
BPM: Beats per minute
CI: Confidence interval
HRR: Heart rate reserve
ICT: Information and communication technology
ITT: Intention-to-treat
LPA: Light physical activity
MET: Metabolic equivalent of task
MMSE: Mini-Mental State Examination
MVPA: Moderate-to-vigorous physical activity
PA: Physical activity
SB: Sedentary behavior
WHO: World Health Organization
Zoom: Zoom video communications
