# Supplementary material for: Effects of frequent, short-duration web-based light-intensity aerobic dance exercise on body composition, physical function, and physical activity in older adults: a randomized controlled trial
Source: BMC Geriatr. 2025 Nov 3;25:831. doi: 10.1186/s12877-025-06495-3 (PMC12581334; doi:10.1186/s12877-025-06495-3)
Supplement: Supplementary file 1 — Supplementary Material 1. [file 12877_2025_6495_MOESM1_ESM.pdf]

Supplementary Material

Effects of frequent, short-duration web-based light-intensity aerobic dance exercise on body composition, physical function, and physical activity in older adults: a randomized controlled trial

Supplementary Figure 1. Changes in the Physical Characteristics and Body Composition Measurements Between Preintervention and Postintervention in the Exercise and Control Groups

Supplementary Figure 2. Changes in the Physical Function Measurements Between Preintervention and Postintervention in the Exercise and Control Groups

Supplementary Figure 3. Changes in the Physical Activity Levels Between Preintervention and Postintervention in the Exercise and Control Groups

Supplementary Table 1. Sensitivity Analysis (Intention-to-Treat, Multiple Imputation): Adjusted Post-Intervention Outcomes (ANCOVA with Baseline Values as Covariates)

Supplementary Table 2. Sensitivity Analysis (Intention-to-Treat): Adjusted Post-Intervention Outcomes (ANCOVA with Baseline Values Plus Step Count as an Additional Covariate)

Supplementary Table 3. Sensitivity Analysis (Per-Protocol): Adjusted Post-Intervention Outcomes (ANCOVA with Baseline Values as Covariates)

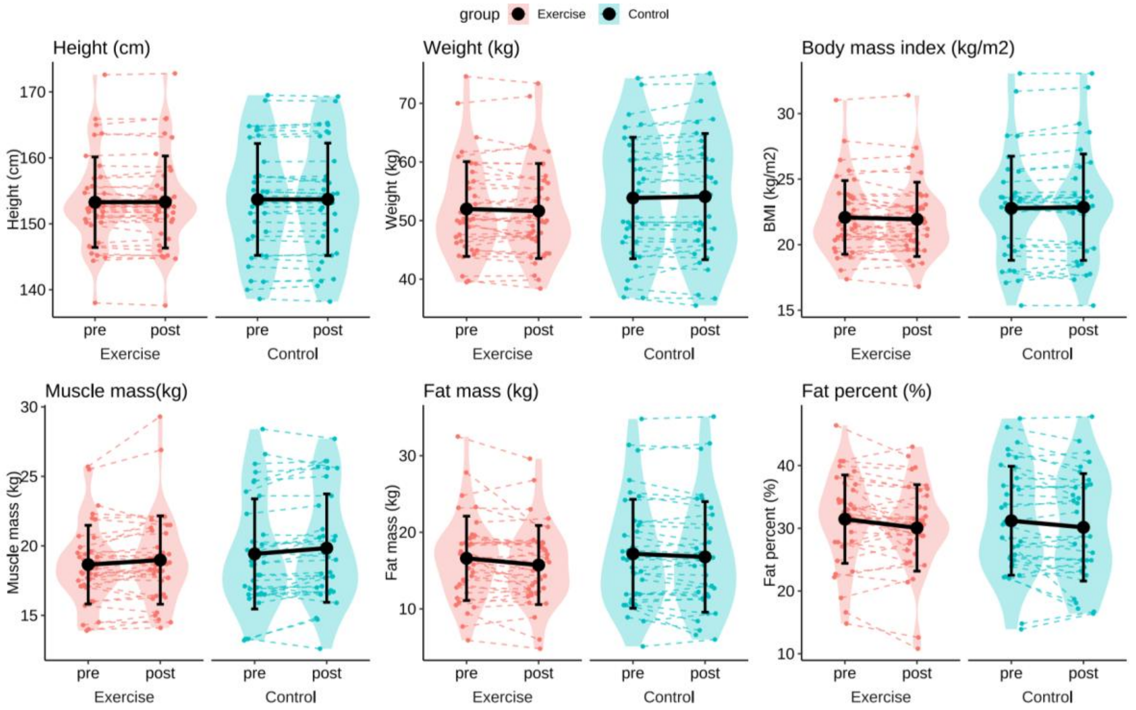

Supplementary Figure 1. Changes in the Physical Characteristics and Body Composition Measurements Between Preintervention and Postintervention in the Exercise and Control Groups

Each dot represents an individual participant, with dashed lines indicating individual changes from pre to post-intervention. Violin plots show the distribution of data. Black circles represent group means and error bars indicate standard deviations.

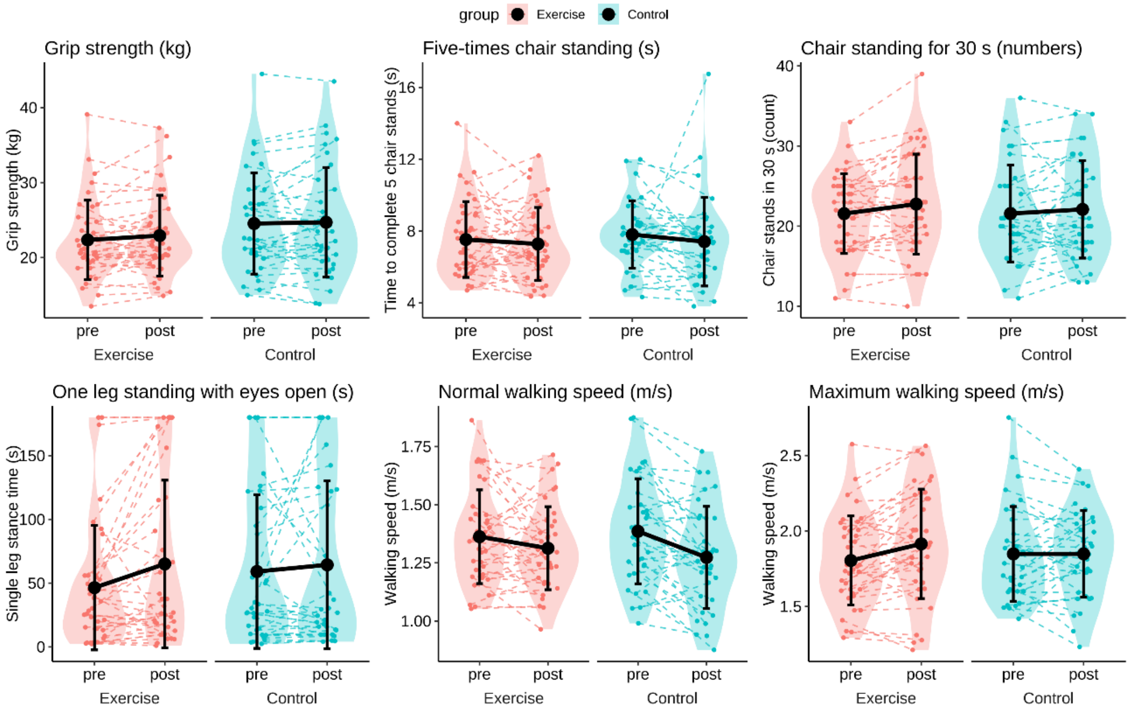

Supplementary Figure 2. Changes in the Physical Function Measurements Between Preintervention and Postintervention in the Exercise and Control Groups

Each dot represents an individual participant, with dashed lines indicating individual changes from pre to post-intervention. Violin plots show the distribution of data. Black circles represent group means and error bars indicate standard deviations.

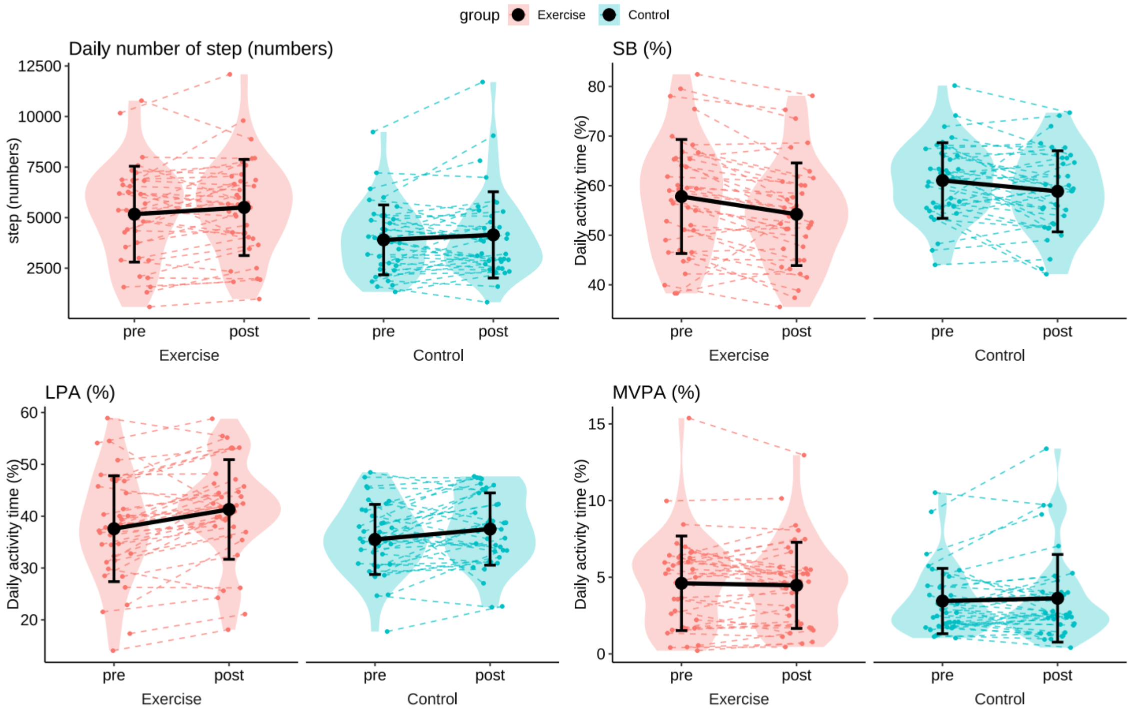

Supplementary Figure 3. Changes in the Physical Activity Levels Between Preintervention and Postintervention in the Exercise and Control Groups.

Each dot represents an individual participant, with dashed lines indicating individual changes from pre to post-intervention. Violin plots show the distribution of data. Black circles represent group means and error bars indicate standard deviations. SB: sedentary behavior, LPA: light physical activity, MVPA: moderate-to-vigorous physical activity.

Supplementary Table 1. Sensitivity Analysis (Intention-to-Treat, Multiple Imputation): Adjusted Post-Intervention Outcomes (ANCOVA with Baseline Values as Covariates)

	Adjusted mean (SE)		Between-group adjusted mean difference (95% CI)	p value	d
	Exercise group (n = 41)	Control group (n = 40)			
Physical characteristics					
Height (cm)	153.6 (0.4)	153.4 (0.5)	0.2 (-1, 1.4)	0.752	0.08
Weight (kg)	53.1 (0.3)	53.6 (0.3)	-0.6 (-1.3, 0.2)	0.133	-0.37
Body mass index (kg/m^2)	22.5 (0.2)	22.7 (0.2)	-0.3 (-0.7, 0.2)	0.226	-0.31
Body composition					
Muscle mass(kg)	19.5 (0.2)	19.5 (0.2)	-0.1 (-0.5, 0.4)	0.753	-0.08
Fat mass (kg)	16.3 (0.3)	16.7 (0.3)	-0.4 (-1.2, 0.4)	0.287	-0.25
Fat percent (%)	30.3 (0.5)	30.5 (0.5)	-0.2 (-1.5, 1.1)	0.731	-0.08
Physical function					
Grip strength (kg)	23.8 (0.5)	23.6 (0.5)	0.2 (-1.2, 1.5)	0.824	0.05
Five-times chair standing (s)	7.3 (0.3)	7.4 (0.3)	-0.1 (-1, 0.9)	0.884	-0.03
Chair standing for 30 s (numbers)	22.7 (0.6)	22 (0.6)	0.7 (-1, 2.4)	0.405	0.2
One leg standing with eyes open (s)	72.6 (7.4)	58.6 (7.5)	14 (-6.9, 35)	0.189	0.31
Normal walking speed (m/s)	1.32 (0.02)	1.27 (0.03)	0.05 (-0.02, 0.12)	0.153	0.34
Maximal walking speed (m/s)	1.93 (0.03)	1.84 (0.03)	0.09 (0, 0.19)	0.053	0.45
Physical activity					
Daily step count (steps/day)	4923.7 (199.9)	4722.7 (198.1)	201 (-350.4, 752.4)	0.475	0.17
SB (%)	55.4 (0.9)	57.5 (0.9)	-2.1 (-4.6, 0.4)	0.103	-0.38
LPA (%)	40.6 (0.8)	38.4 (0.8)	2.3 (0, 4.5)	0.048*	0.46
MVPA (%)	4 (0.2)	4.2 (0.2)	-0.2 (-0.9, 0.4)	0.52	-0.15

Note. Using mice package (version 3.17) in R, we used multiple imputation by chained equations to impute missings data on the outcome using the predictive mean matching. Fifty imputed datasets were generated, and results were pooled according to Rubin's rules (van Buuren & Groothuis-Oudshoorn, 2011).
SB: sedentary behavior, LPA: light physical activity, MVPA: moderate-to-vigorous physical activity. *p < 0.05.
d = Cohen's d.

Reference: van Buuren S, Groothuis-Oudshoorn K. Mice: Multivariate imputation by chained equations in R. J Stat Softw. 2011;45:3. doi:10.18637/ jss.v045.i03

Supplementary Table 2. Sensitivity Analysis (Intention-to-Treat): Adjusted Post-Intervention Outcomes (ANCOVA with Baseline Values Plus Step Count as an Additional Covariate)

	Exercise group		Control group		Between-group adjusted mean difference (95% CI)	p value	d
	N	Adjusted mean (SE)	N	Adjusted mean (SE)			
Physical characteristics							
Height (cm)	37	153.6 (0.1)	34	153.6 (0.1)	0 (-0.2, 0.2)	0.906	0.03
Weight (kg)	37	52.7 (0.2)	34	53.3 (0.2)	-0.7 (-1.4, 0)	0.063	-0.48
Body mass index (kg/m^2)	37	22.3 (0.1)	34	22.6 (0.1)	-0.3 (-0.6, 0)	0.093	-0.43
Body composition							
Muscle mass(kg)	36	19.3 (0.2)	34	19.5 (0.2)	-0.2 (-0.6, 0.3)	0.409	-0.21
Fat mass (kg)	36	16.1 (0.3)	34	16.6 (0.3)	-0.4 (-1.2, 0.4)	0.317	-0.26
Fat percent (%)	36	30.2 (0.5)	34	30.3 (0.5)	-0.1 (-1.5, 1.3)	0.887	-0.04
Physical function							
Grip strength (kg)	37	24.1 (0.5)	34	23.6 (0.5)	0.5 (-1, 2)	0.501	0.17
Five-times chair standing (s)	37	7.4 (0.3)	34	7.3 (0.4)	0.1 (-0.9, 1.1)	0.883	0.04
Chair standing for 30 s (numbers)	37	23 (0.6)	33	22 (0.7)	0.9 (-1, 2.8)	0.342	0.24
One leg standing with eyes open (s)	37	68.3 (7.6)	34	62.6 (7.9)	5.8 (-16.8, 28.4)	0.61	0.13
Normal walking speed (m/s)	37	1.32 (0.02)	34	1.27 (0.03)	0.06 (-0.02, 0.13)	0.123	0.4
Maximal walking speed (m/s)	37	1.94 (0.03)	34	1.83 (0.04)	0.11 (0.01, 0.21)	0.03*	0.57
Physical activity							
Daily step count (steps/day)	37	4906.5 (194.9)	37	4743.4 (194.9)	163.1 (-399, 725.2)	0.565	0.14
SB (%)	37	55.8 (0.9)	37	57.3 (0.9)	-1.5 (-4, 1.1)	0.264	-0.27
LPA (%)	37	40.3 (0.8)	37	38.5 (0.8)	1.8 (-0.6, 4.2)	0.147	0.36
MVPA (%)	37	3.9 (0.2)	37	4.2 (0.2)	-0.3 (-1, 0.4)	0.372	-0.22

Note. SB: sedentary behavior, LPA: light physical activity, MVPA: moderate-to-vigorous physical activity. *$p < 0.05$. d = Cohen's d.

Supplementary Table 3. Sensitivity Analysis (Per-Protocol): Adjusted Post-Intervention Outcomes (ANCOVA with Baseline Values as Covariates)

	Exercise group		Control group		Between-group adjusted mean difference (95% CI)	p value	d
	N	Adjusted mean (SE)	N	Adjusted mean (SE)			
Physical characteristics							
Height (cm)	35	153.8 (0.1)	31	153.8 (0.1)	0.1 (-0.1, 0.3)	0.448	0.19
Weight (kg)	35	52.9 (0.2)	31	53.5 (0.3)	-0.6 (-1.3, 0.1)	0.084	-0.44
Body mass index (kg/m^2)	35	22.3 (0.1)	31	22.6 (0.1)	-0.3 (-0.6, 0)	0.088	-0.43
Body composition							
Muscle mass(kg)	34	19.5 (0.2)	31	19.6 (0.2)	0 (-0.5, 0.4)	0.822	-0.06
Fat mass (kg)	34	16.1 (0.3)	31	16.7 (0.3)	-0.6 (-1.4, 0.2)	0.161	-0.35
Fat percent (%)	34	29.9 (0.5)	31	30.3 (0.5)	-0.4 (-1.8, 0.9)	0.524	-0.16
Physical function							
Grip strength (kg)	35	24 (0.5)	31	24.1 (0.5)	0 (-1.5, 1.5)	0.963	-0.01
Five-times chair standing (s)	35	7.3 (0.3)	31	7.3 (0.4)	0.1 (-1, 1.1)	0.918	0.03
Chair standing for 30 s (numbers)	35	22.9 (0.6)	30	22.4 (0.7)	0.5 (-1.4, 2.4)	0.593	0.13
One leg standing with eyes open (s)	35	73.9 (7.8)	31	62.2 (8.3)	11.6 (-11.4, 34.6)	0.316	0.25
Normal walking speed (m/s)	35	1.33 (0.02)	31	1.26 (0.03)	0.06 (-0.01, 0.13)	0.085	0.43
Maximal walking speed (m/s)	35	1.95 (0.03)	31	1.85 (0.04)	0.1 (0, 0.2)	0.04*	0.52
Physical activity							
Daily step count (steps/day)	35	4935.4 (195.2)	34	4733.5 (198.2)	201.8 (-365.4, 769.1)	0.48	0.18
SB (%)	35	55.9 (0.9)	34	58.2 (0.9)	-2.3 (-4.9, 0.4)	0.09	-0.42
LPA (%)	35	40.1 (0.8)	34	37.9 (0.9)	2.2 (-0.2, 4.6)	0.071	0.45
MVPA (%)	35	3.9 (0.2)	34	4.1 (0.2)	-0.2 (-0.9, 0.5)	0.605	-0.13

Note. SB: sedentary behavior, LPA: light physical activity, MVPA: moderate-to-vigorous physical activity. *$p < 0.05$. d = Cohen's d.
